# Impact of lumbar pedicle subtraction osteotomy (PSO) level on global alignment and proportion (GAP) score in revision adult iatrogenic flatback spinal deformities

**DOI:** 10.1007/s43390-025-01141-9

**Published:** 2025-07-08

**Authors:** Perry Lim, Aaron J. Clark, Austen D. Katz, Asra Toobaie, Vedat Deviren, Christopher P. Ames, Alekos A. Theologis

**Affiliations:** 1 Department of Orthopedic Surgery, University of California - San Francisco, 500 Parnassus Ave, MUW 3 Floor, San Francisco, CA 94143 USA; 2 Department of Neurological Surgery, UCSF, San Francisco, CA USA

**Keywords:** Adult spinal deformity, Iatrogenic flatback, Lumbar pedicle subtraction osteotomy, GAP score, Mechanical failures

## Abstract

**Purpose:**

To explore the impact of different lumbar pedicle subtraction osteotomy (L-PSO) levels on Global Alignment and Proportion (GAP) scores.

**Methods:**

Adults at a single center who underwent lumbar PSOs with revision instrumentation [thoracolumbar junction (T9-L1) to pelvis] and a minimum 2-year follow-up were reviewed. The patients were divided by level of PSO (L2, L3, L4, and L5) and compared with respect to demographic and surgical data, sagittal parameters, GAP scores, and mechanical complications requiring revision operations.

**Results:**

152 patients (average age 64.4 ± 10.6 years, average follow-up 9.0 ± 4.1 years) were included for analysis. L3 (40.8%) and L4 (45.4%) PSOs were more common than L2 (4.6%) and L5 (9.2%) PSOs. Average pre-op GAP scores (9.8 ± 2.8) were similar and improved significantly for all L-PSO levels, although post-op GAP scores (7.1 ± 2.2) remained “disproportioned” for all L-PSO levels. Post-op Lumbar Distribution Index (LDI) scores were significantly better for lower PSOs (L4 + L5) given better improvement of L4-S1 lordosis. The revisions for mechanical failures were higher in L2 and L3 PSOs. Average post-op GAP scores were not different for patients who did and did not undergo mechanical failure revisions.

**Conclusions:**

L-PSOs, irrespective of the level, improve GAP scores. While residual disproportionate post-operative alignment was observed for all L-PSO levels, distal PSOs improved L4-S1 lordosis and LDI scores to a greater extent than proximal PSOs. Although more distal lumbar PSOs also had lower rates of revision operations for mechanical complications, other patient and surgical factors also likely played a role in the observed rates of mechanical failures.

## Introduction

The first lumbar pedicle subtraction osteotomy (L-PSO) was performed by Eivind Thomasen in 1985 [1]. Thomasen’s PSO was performed at the L2 level and resulted in 12–50 degrees (average 28.3 degrees) of segmental lordosis correction [1]. Since that time, considerable advancements have occurred with respect to L-PSO’s surgical technique [2] and a clearer understanding of their corrective potential and utility (radiographic and functional) [3–10], complication profiles [11–14], and optimized biomechanical stabilization strategies [15–22]. Of persistent debate remains the ideal location at which a L-PSO should be performed to optimize sagittal alignment [9, 23]. To this end, there is particularly limited information on the effects of L-PSOs on the GAP score [23, 24], an important and validated scoring strategy of sagittal alignment to minimize mechanical complications in ASD operations [25–31]. As such, the aim of this retrospective cohort analysis is to evaluate and compare changes in the GAP score and associated mechanical complications between PSOs performed at 4 different levels in the lumbar spine for revision adult thoracolumbar spinal deformities.

## Methods

**Fig. 1:**
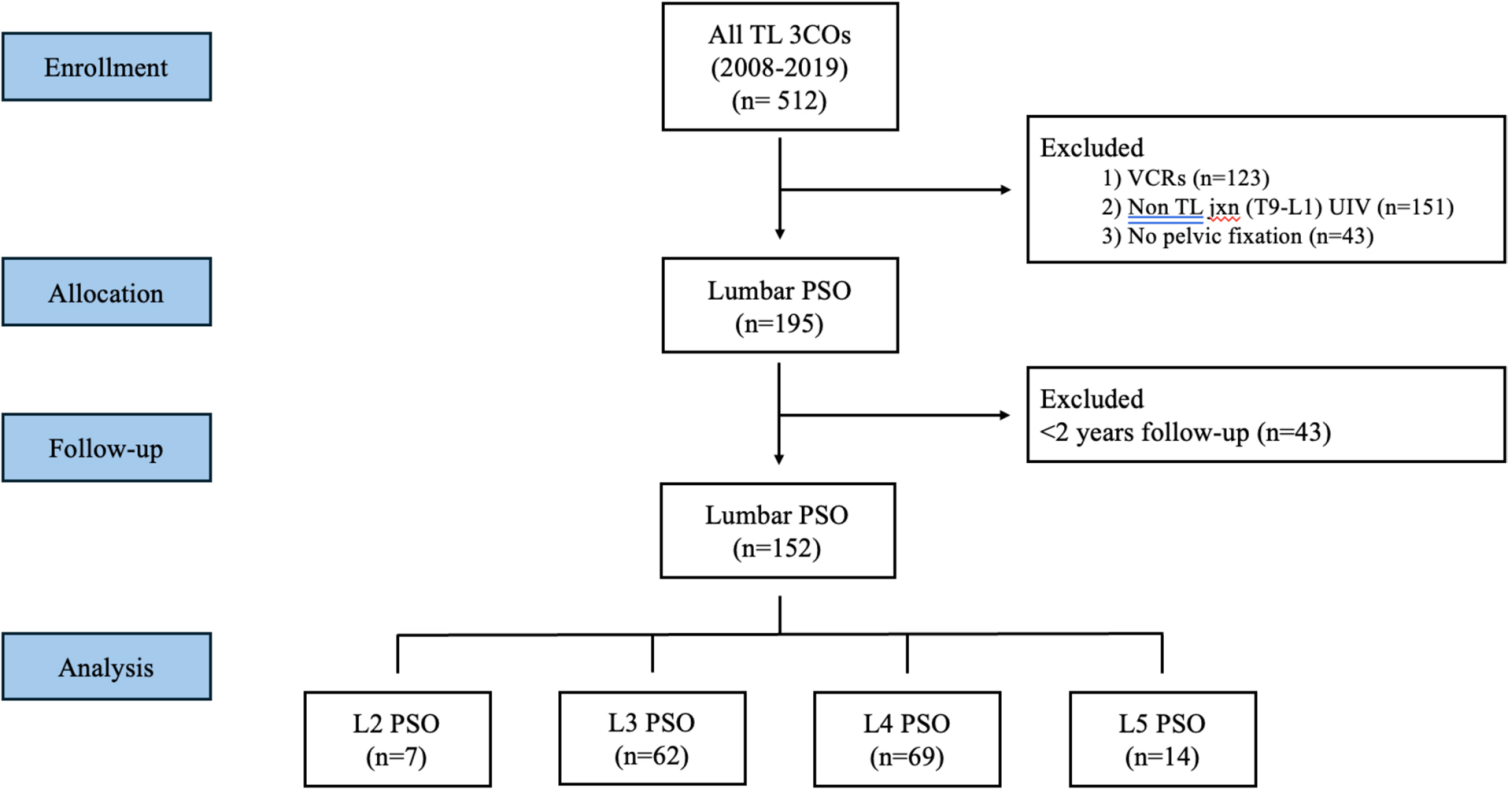
CONSORT flow chart of included patients. *TL = thoracolumbar; Jxn = junction; 3CO = 3-column osteotomy; VCR = vertebral column resection; PSO = pedicle subtraction osteotomy; UIV = upper instrumented vertebrae

After institutional board review approval, consecutive adults at a single academic medical center who had undergone a prior lumbar PSO by two pairs/teams of surgeons for iatrogenic lumbar flatback deformity between 2008 and 2019 were identified and their charts were reviewed for data as outlined below. The inclusion criteria included single-level lumbar PSO at L2, L3, L4, or L5, revision posterior instrumented fusion from the thoracolumbar junction (TLJxn; T9-L1) to the pelvis, circumferential fusions of the lumbar spine, and minimum post-operative follow-up of 2 years (Fig. 1). All operations were performed using 5.5 mm cobalt chrome rods as the primary midline rods and 5.5 mm titanium rods as accessory rods and/or satellite rods (in-line or lateral) spanning the L-PSO site if applicable, traditional iliac screws, without supplemental intradiscal techniques, and with low-dose bone morphogenetic protein (1–2 large kits), morselized crushed cancellous allograft, and local autograft bone to augment the posterior fusion at all levels. No interbody cages were used within the PSO or adjacent to the PSO at the cranial level, as all PSOs were considered Schwab Type IV osteotomies. For the caudal disk adjacent to the PSO, a transforaminal lumbar interbody cage was inserted if there was no prior interbody support. If there was prior interbody support at the caudal disk, no additional interbody support was attempted. The excluded patients included those with ankylosing spondylitis, pathological fractures from infection or tumors, neuromuscular disorders (i.e., Parkinson’s disease, multiple sclerosis, myopathies), and prior instrumented fusions isolated to the cervical or thoracic spine, and those with less than 2 years of post-operative follow-up.

### Data variables

The demographic variables collected included age, sex, body mass index (BMI), and last date of follow-up. The operative variables encompassed levels instrumented and level of PSO, number of rods, and number of points of pelvic fixation. Perioperative outcomes, including reoperations, rod fractures, and proximal junctional kyphosis/proximal junctional failure (PJK/PJF), were recorded. The presence of rod fractures and PJK/PJF were assessed on each post-operative radiograph. The radiographic alignment data were evaluated on pre-operative and 3-month postoperative full-length free-standing postero-anterior and lateral spine radiographs. Three-month post-operative radiographs were used so as to capture the change from pre-op to post-op alignment most closely by minimizing the influence of loss of sagittal alignment in the post-operative setting that often occurs over time [32]. Radiographic alignment measurements consisted of the global tilt (GT), L1–S1 lordosis, L4–S1 lordosis, pelvic incidence (PI), sacral slope (SS), pelvic tilt (PT), thoracic kyphosis (TK), L1 pelvic angle (L1PA), T4 pelvic angle (T4PA), and PSO angle (as defined as the endplate cranial to the PSO to the endplate of caudal to the PSO level—i.e., for a L3 PSO, the PSO angle was defined as the angle between the L2 caudal endplate and the L3 caudal endplate). All radiographic measurements were conducted by an experienced spine surgeon using the PACS imaging system of our university’s medical record system.

### GAP score calculation

Utilizing the previously mentioned radiographic measurements, the total GAP score and its subdomains were derived, including relative pelvic version (RPV), lordosis distribution index (LDI), relative lumbar lordosis (RLL), and relative spinopelvic alignment (RSA) [25]. Additionally, “ideal” values were calculated from measured radiographic data to include in the final GAP score [25]. Raw and ideal radiographic values were used to calculate the 5 components of the validated GAP score, as previously described by Yilgor et al. [25]. Final GAP scores were then stratified into one of three spinopelvic groups: proportioned (GAP score = 0–2), moderately disproportioned (GAP score = 3–6), or severely proportioned (GAP score ≥ 7), as previously described by Yilgor et al. [25]. A higher total GAP score as well as higher values in each of the GAP’s subdomains signify worsening sagittal malalignment [25].

### Statistical analysis

The patients were stratified by level of PSO (L2, L3, L4, or L5) and the aforementioned demographic information and radiographic data were compared. The data were determined to have a normal distribution based on histograms. Descriptive statistical analysis was performed, which included the mean ± standard deviation for continuous variables and the percentage for categorical variables. The categorical variables were compared using a chi-squared test. Continuous variables were compared using one-way analysis of variant tests. Statistical significance was set at *p* < 0.05. R Studio (version 4.3.1, Vienna, Austria) was used to perform the statistical analyses.

## Results

### Patient cohort

The baseline demographic data are presented in Table 1. A total of 152 patients met inclusion criteria and were analyzed. Mean age at the index operation was 64.4 ± 10.6 years with 43% were females. The mean BMI was 29.7 ± 5.8 kg/m^2^ and the average length of follow up was 9.0 ± 4.1 years. Distribution of sex was significantly different between the PSO groups with the L2 PSO group having a higher percentage of females and the L4 and L5 PSO groups having a higher percentage of males. The average ages and BMI were similar between the four different PSO groups. There were significant differences in number of rods and number of points of pelvic fixation between the PSO groups with L4 and L5 PSOs having significantly more constructs consisting of multiple rods (≥ 4) and 2 iliac screws in each hemipelvis (i.e., four pelvic screws total). Average follow-up was significantly different between the 4 PSO groups with patients with L2 PSO (12.4 ± 1.8) and L3 PSOs (11.1 ± 3.3) being significantly longer than the L4 (7.5 ± 3.8) and L5 PSO (5.8 ± 3.6) patients. The majority of PSOs were at the L3 (*n* = 62; 40.8%) and L4 level (*n* = 69; 45.4%), with the minority being at L2 (*n* = 7; 4.6%) and L5 (*n* = 14; 9.2%).

**Table 1:** Patient demographics

Characteristics	Total cohort (*n* = 152)	L2 PSO (*n* = 7)	L3 PSO (*n* = 62)	L4 PSO (*n* = 69)	L5 PSO (*n* = 14)	*P*
Age (years)	64.4 ± 10.6	64.5 ± 10.0	63.1 ± 11.0	65.7 ± 10.5	63.4 ± 10.5	0.55
Sex						**0.049**
Male	86 (57%)	2 (29%)	29 (47%)	46 (67%)	9 (64%)	
Female	66 (43%)	5 (71%)	33 (53%)	23 (33%)	5 (36%)	
BMI (kg/m^2^)	29.7 ± 5.8	30.6 ± 6.9	29.9 ± 5.8	29.7 ± 5.7	28.3 ± 6.2	0.80
Follow up (years)	9.0 ± 4.1	12.4 ± 1.8	11.1 ± 3.3	7.5 ± 3.8	5.8 ± 3.6	**< 0.001**
# Rods						
2	51 (34%)	6 (86%)	32 (52%)	12 (17%)	1 (7%)	**< 0.001**
3	2 (1%)	0 (0%)	1 (2%)	1 (1%)	0 (0%)	0.95
4	58 (38%)	1 (14%)	27 (44%)	25 (36%)	5 (36%)	0.46
> 4	41 (27%)	0 (0%)	2 (3%)	31 (45%)	8 (57%)	**< 0.001**
Average ± SD	3.8 ± 1.5	2.3 ± 0.8	3 ± 1.1	4.5 ± 1.5	5 ± 1.4	**< 0.001**
# Pelvic screws						
1	5 (3%)	1 (14%)	4 (6%)	0 (0%)	0 (0%)	0.059
2	104 (68%)	6 (86%)	55 (89%)	40 (58%)	3 (21%)	**< 0.001**
3	8 (5%)	0 (0%)	2 (3%)	4 (6%)	2 (14%)	0.36
4	35 (23%)	0 (0%)	1 (2%)	25 (36%)	9 (64%)	**< 0.001**
Average ± SD	2.5 ± 0.9	1.88 ± 0.4	2 ± 0.4	2.8 ± 1.0	3.4 ± 0.9	**< 0.001**

*p* < 0.05 defines statistical significance (in bold)

*BMI = body mass index; L = lumbar; PSO = pedicle subtraction osteotomy; SD = standard deviation

### Sagittal spinal alignment parameters

Data for sagittal spinal alignment are presented in Table 2. Lumbopelvic mismatch and associated compensatory mechanisms were present for the entire cohort pre-operatively, evidenced by loss of L1-S1 lordosis, decreased L4-S1 lordosis, and increased pelvic tilt. All pre-operative sagittal alignment parameters were similar between the four PSO groups except the L4 and L5 PSO groups had significantly less L4-S1 lordosis pre-operatively and the total L1-S1 pre-operative lordosis was significantly greater for the L5 PSO patients. L3 and L4 PSOs were found to have significantly higher segmental PSO correction, as evidenced by higher PSO angles. For all PSO groups, all sagittal alignment parameters improved after surgery. There were no differences in average post-operative sagittal alignment parameters between the four groups, except for L4-S1 lordosis, which was significantly higher in the L4 and L5 PSO cohorts as a result of the lower PSOs (L4 and L5) creating significantly greater improvements in L4-S1 lordosis than more proximal PSOs (L2 and L3).

**Table 2:** Sagittal spinal alignment radiographic parameters

Parameter	Total (*n* = 152)	L2 PSO (*n* = 7)	L3 PSO (*n* = 62)	L4 PSO (*n* = 69)	L5 PSO (*n* = 14)	*P*
PI (degrees)	62.3 ± 15.5	57.6 ± 14.5	64.5 ± 14.9	59.6 ± 16.4	68.2 ± 12.2	0.11
PT (degrees)						
Preop	29.5 ± 10.6	26.3 ± 5.2	28.7 ± 9.7	29.6 ± 12.2	34.0 ± 6.6	0.32
Postop	24.5 ± 16.4	22.6 ± 15.8	24.2 ± 16.7	24.0 ± 17.0	29.5 ± 13.0	0.69
Delta	− 5.0 ± 13.5	− 3.7 ± 13.9	− 4.5 ± 15.2	− 5.6 ± 12.8	− 4.5 ± 9.2	0.97
SS (degrees)						
Preop	32.8 ± 14.0	31.3 ± 11.8	35.7 ± 13.5	30.1 ± 15.4	34.2 ± 7.2	0.14
Postop	37.8 ± 12.8	35.0 ± 5.7	40.3 ± 13.4	35.6 ± 13.6	38.7 ± 5.0	0.2
Delta	5.0 ± 13.5	3.7 ± 13.9	4.5 ± 15.2	5.6 ± 12.8	4.5 ± 9.2	0.97
L4-S1 lordosis (degrees)						
Preop	26.3 ± 13.2	34.4 ± 10.8	29.3 ± 13.6	23.3 ± 12.3	23.4 ± 13.4	**0.018**
Postop	34.2 ± 16.7	29.1 ± 9.1	25.6 ± 14.0	41.4 ± 15.6	38.7 ± 18.8	**< 0.001**
Delta	7.9 ± 19.7	− 5.3 ± 15.2	− 3.7 ± 15.9	18.1 ± 17.1	15.3 ± 17.9	**< 0.001**
L1-S1 lordosis (degrees)						
Preop	32.7 ± 16.6	32.9 ± 22.9	34.7 ± 15.3	29.0 ± 16.5	42.6 ± 15.7	**0.025**
Postop	56.0 ± 13.3	54.4 ± 11.8	58.6 ± 14.8	53.5 ± 12.3	57.8 ± 10.3	0.17
Delta	23.3 ± 17.2	21.6 ± 25.0	23.9 ± 17.6	24.5 ± 16.4	15.2 ± 14.5	0.31
TK (degrees)						
Preop	22.5 ± 13.2	21.0 ± 10.6	22.3 ± 13.3	21.7 ± 13.4	27.5 ± 13.4	0.51
Postop	39.4 ± 13.7	42.4 ± 9.0	39.4 ± 15.0	38.5 ± 12.4	42.9 ± 15.7	0.67
Delta	17.0 ± 13.5	21.4 ± 8.0	17.0 ± 14.6	16.7 ± 13.7	15.4 ± 9.0	0.81
Global Tilt (degrees)						
Preop	39.8 ± 12.1	32.1 ± 6.9	38.3 ± 11.6	41.4 ± 13.0	42.9 ± 10.0	0.12
Postop	26.4 ± 10.2	23.4 ± 12.6	26.5 ± 10.4	26.2 ± 10.3	28.6 ± 8.3	0.75
Delta	− 13.4 ± 13.0	− 8.7 ± 16.0	− 11.8 ± 12.0	− 15.1 ± 13.6	− 14.3 ± 13.2	0.38
L1 PA (degrees)						
Preop	24.1 ± 10.1	12.6 ± 6.9	19.5 ± 7.6	27.8 ± 8.6	31.9 ± 14.1	**< 0.01**
Postop	18.4 ± 8.9	12.9 ± 6.5	14.7 ± 6.8	21.2 ± 8.5	23.4 ± 12.6	**< 0.01**
Delta	− 5.7 ± 7.0	0.3 ± 2.8	− 4.8 ± 5.4	− 6.6 ± 7.7	− 8.4 ± 9.1	**0.02**
T4 PA (degrees)						
Preop	31.9 ± 11.2	24.1 ± 7.7	26.4 ± 8.7	36.3 ± 10.6	38.5 ± 13.3	**< 0.01**
Postop	21.0 ± 9.8	14.9 ± 5.8	16.0 ± 7.6	24.7 ± 9.1	27.5 ± 11.7	**< 0.01**
Delta	− 10.9 ± 9.2	− 9.3 ± 9.6	− 10.4 ± 8.2	− 11.6 ± 10.2	− 11.0 ± 8.7	0.86
T4-L1 PA mismatch (degrees)						
Preop	7.8 ± 6.2	11.6 ± 7.8	7.0 ± 6.0	8.5 ± 6.3	6.6 ± 5.6	0.18
Postop	2.6 ± 4.4	2.0 ± 3.3	1.4 ± 4.5	3.5 ± 4.3	4.1 ± 4.0	**0.02**
Delta	− 5.2 ± 5.4	− 9.6 ± 8.4	− 5.6 ± 5.0	− 5.0 ± 5.4	− 2.6 ± 4.5	**0.04**
PSO angle (degrees)	27.6 ± 7.6	23.9 ± 6.8	29.1 ± 6.4	27.6 ± 7.5	22.8 ± 10.5	**0.02**

*p* < 0.05 defines statistical significance (in bold)

PI = pelvic incidence; PT = pelvic tilt; SS = sacral slope; TK = thoracic kyphosis; PA = pelvic angle; PSO = pedicle subtraction osteotomy

### GAP proportional alignment domain scores

Data for calculated GAP scores within each of its domains are presented in Table 3. Total GAP scores pre-operatively were similar between all 4 PSO levels. For RPV, RLL, and RSA subscores, all four PSO level cohorts had significantly similar pre-operative values as well as similar improvements after surgery with resultant similar post-operative values. For the LDI subscore, the L4 and L5 PSO cohorts were noted to have significantly higher LDI scores post-operatively compared to the L2 and L3 PSO cohorts, which was a result of the former two groups having significantly greater relative change in the LDI score from pre-op to post-op.

**Table 3:** GAP domain proportional radiographic parameter scores

GAP scores	Total (*n* = 152)	L2 PSO (*n* = 7)	L3 PSO (*n* = 62)	L4 PSO (*n* = 69)	L5 PSO (*n* = 14)	*p*
Total GAP score						
Preop	9.8 ± 2.8	9.4 ± 2.9	9.5 ± 3.0	10.0 ± 2.7	9.9 ± 1.8	0.82
Postop	7.1 ± 2.2	6.3 ± 2.1	6.8 ± 2.2	7.5 ± 2.4	7.3 ± 1.4	0.29
Delta	− 2.6 ± 3.2	− 3.1 ± 3.9	− 2.7 ± 3.5	− 2.5 ± 3.0	− 2.6 ± 2.2	0.95
RPV subscore						
Preop	2.0 ± 1.1	2.0 ± 1.0	1.7 ± 1.3	2.1 ± 1.1	2.4 ± 0.5	0.06
Postop	1.4 ± 1.2	1.4 ± 1.1	1.5 ± 1.1	1.4 ± 1.3	1.5 ± 1.2	0.94
Delta	− 0.5 ± 1.6	− 0.6 ± 1.8	− 0.2 ± 1.7	− 0.8 ± 1.5	− 0.9 ± 1.3	0.17
LDI subscore						
Preop	1.9 ± 1.3	2.1 ± 1.5	2.1 ± 1.2	1.7 ± 1.4	1.6 ± 1.3	0.17
Postop	1.5 ± 1.2	1.0 ± 1.1	1.1 ± 0.9	1.9 ± 1.4	1.9 ± 1.3	**0.002**
Delta	− 0.3 ± 1.8	− 1.1 ± 1.9	− 1.0 ± 1.6	0.2 ± 1.8	0.3 ± 1.8	**< 0.001**
RLL subscore						
Preop	2.8 ± 0.4	2.6 ± 0.5	2.8 ± 0.4	2.9 ± 0.3	2.7 ± 0.5	0.11
Postop	2.2 ± 0.4	2.1 ± 0.4	2.3 ± 0.5	2.2 ± 0.4	2.3 ± 0.5	0.5
Delta	− 0.6 ± 0.5	− 0.4 ± 0.5	− 0.5 ± 0.6	− 0.7 ± 0.5	− 0.4 ± 0.5	0.11
RSA subscore						
Preop	2.4 ± 1.0	2.0 ± 1.3	2.2 ± 1.1	2.6 ± 0.9	2.7 ± 0.7	0.08
Postop	1.2 ± 1.2	1.0 ± 1.4	1.1 ± 1.3	1.3 ± 1.2	1.1 ± 1.1	0.85
Delta	− 1.2 ± 1.4	− 1.0 ± 1.8	− 1.1 ± 1.4	− 1.3 ± 1.3	− 1.6 ± 1.5	0.57

*p* < 0.05 defines statistical significance (in bold)

GAP = global alignment and proportion; L = lumbar; PSO = pedicle subtraction osteotomy; RPV = relative pelvic version; LDI = lordosis distribution index; RLL = relative lumbar lordosis; RSA = relative spinopelvic alignment

### GAP descriptive classifications

The descriptive categorization of alignment for total GAP score (proportioned, moderately disproportioned, severely disproportioned) as well as for the GAP’s subdomains are presented in Table 4. For the overall cohort, there was a significant improvement between the pre-operative and post-operative descriptive classifications for the GAP score and all its subscores (RPV, LDI, RLL, and RSA). Specifically, significant pre-operative to post-operative shifts from severely disproportioned (84% to 57%) to moderately disproportioned (16–43%) (*p* < 0.001) were found for all patients.

**Table 4:** GAP descriptive classifications/categories

	Total (*n* = 152)	L2 PSO (*n* = 7)	L3 PSO (*n* = 62)	L4 PSO (*n* = 69)	L5 PSO (*n* = 14)	*P*
*Total GAP score*						
Preop						
Proportioned	1 (1%)	0 (0%)	1 (2%)	0 (0%)	0 (0%)	0.69
Moderately disproportioned	24 (16%)	2 (29%)	12 (19%)	9 (13%)	1 (7%)	0.45
Severely disproportioned	127 (84%)	5 (71%)	49 (79%)	60 (87%)	13 (93%)	0.37
Postop						
Proportioned	1 (1%)	0 (0%)	0 (0%)	1 (1%)	0 (0%)	0.75
Moderately disproportioned	65 (43%)	5 (71%)	30 (48%)	27 (39%)	3 (21%)	0.11
Severely disproportioned	86 (57%)	2 (29%)	32 (52%)	41 (59%)	11 (79%)	0.12
Preop versus Postop *p*-value	**< 0.001**	0.11	**0.002**	**0.001**	0.28	
*RPV*						
Preop						
Anteversion	5 (3%)	0 (0%)	2 (3%)	3 (4%)	0 (0%)	0.81
Aligned	32 (21%)	1 (14%)	20 (32%)	11 (16%)	0 (0%)	**0.021**
Moderate retroversion	50 (33%)	4 (57%)	17 (27%)	21 (30%)	8 (57%)	0.08
Severe retroversion	65 (43%)	2 (29%)	23 (37%)	34 (49%)	6 (43%)	0.46
Postop						
Anteversion	23 (15%)	1 (14%)	14 (23%)	8 (12%)	0 (0%)	0.12
Aligned	50 (33%)	2 (29%)	16 (26%)	27 (39%)	5 (36%)	0.43
Moderate retroversion	41 (27%)	3 (43%)	17 (27%)	15 (22%)	6 (43%)	0.30
Severe retroversion	38 (25%)	1 (14%)	15 (24%)	19 (28%)	3 (21%)	0.85
Preop versus Postop *p*-value	**< 0.001**	0.61	**0.011**	**0.002**	**0.043**	
*LDI*						
Preop						
Hyperlordotic maldistribution	80 (53%)	5 (71%)	39 (63%)	32 (46%)	4 (29%)	**0.047**
Aligned	45 (30%)	2 (29%)	13 (21%)	25 (36%)	5 (36%)	0.27
Moderate hypolordotic maldistribution	10 (7%)	0 (0%)	5 (8%)	5 (7%)	0 (0%)	0.63
Severe hypolordotic maldistribution	17 (11%)	0 (0%)	5 (8%)	7 (10%)	5 (36%)	**0.018**
Postop						
Hyperlordotic maldistribution	47 (31%)	1 (14%)	2 (3%)	38 (55%)	6 (43%)	**< 0.001**
Aligned	49 (32%)	3 (43%)	21 (34%)	21 (30%)	4 (29%)	0.89
Moderate hypolordotic maldistribution	21 (14%)	2 (29%)	14 (23%)	5 (7%)	0 (0%)	**0.018**
Severe hypolordotic maldistribution	35 (23%)	1 (14%)	25 (40%)	5 (7%)	4 (29%)	**< 0.001**
Preop versus Postop *p*-value	**< 0.001**	0.12	**< 0.001**	0.75	0.73	
*RLL*						
Preop						
Hyperlordosis	0 (0%)	0 (0%)	0 (0%)	0 (0%)	0 (0%)	N/A
Aligned	0 (0%)	0 (0%)	0 (0%)	0 (0%)	0 (0%)	N/A
Moderate hypolordosis	28 (18%)	3 (43%)	13 (21%)	8 (12%)	4 (29%)	0.10
Severe hypolordosis	124 (82%)	4 (57%)	49 (79%)	61 (88%)	10 (71%)	0.10
Postop						
Hyperlordosis	5 (3%)	1 (14%)	3 (5%)	1 (1%)	0 (0%)	0.23
Aligned	0 (0%)	0 (0%)	0 (0%)	0 (0%)	0 (0%)	N/A
Moderate hypolordosis	116 (76%)	6 (86%)	44 (71%)	56 (81%)	10 (71%)	0.49
Severe hypolordosis	31 (20%)	0 (0%)	15 (24%)	12 (17%)	4 (29%)	0.35
Preop versus Postop *p*-value	**< 0.001**	**0.049**	**< 0.001**	**< 0.001**	**0.023**	
*RSA*						
Preop						
Negative malignment	0 (0%)	0 (0%)	0 (0%)	0 (0%)	0 (0%)	N/A
Aligned	12 (8%)	1 (14%)	6 (10%)	5 (7%)	0 (0%)	0.59
Moderate positive malalignment	27 (18%)	2 (29%)	16 (26%)	7 (10%)	2 (14%)	0.10
Severe positive malalignment	113 (74%)	4 (57%)	40 (65%)	57 (83%)	12 (86%)	0.05
Postop						
Negative malignment	6 (4%)	0 (0%)	2 (3%)	3 (4%)	1 (7%)	0.85
Aligned	60 (39%)	4 (57%)	31 (50%)	21 (30%)	4 (29%)	0.08
Moderate positive malalignment	40 (26%)	1 (14%)	9 (15%)	24 (35%)	6 (43%)	**0.023**
Severe positive malalignment	46 (30%)	2 (29%)	20 (32%)	21 (30%)	3 (21%)	0.89
Preop versus Postop *p*-value	**< 0.001**	0.25	**< 0.001**	**< 0.001**	**0.006**	

*p* < 0.05 defines statistical significance (in bold)

GAP = global alignment and proportion; L = lumbar; PSO = pedicle subtraction osteotomy; RPV = relative pelvic version; LDI = lordosis distribution index; RLL = relative lumbar lordosis; RSA = relative spinopelvic alignment

When comparing the four different PSO level cohorts, there were no differences in the descriptive subclassifications pre-operatively and post-operatively for the total GAP score. For the GAP’s subdomains pre-operatively, there were statistical differences in the percentage of “aligned” patients for the RPV and for “hyperlordotic maldistribution” and “severe hypolordotic maldistribution” patients for the LDI. For the GAP’s subdomains post-operatively, there were statistical differences in the percentage of “moderate positive malalignment” patients for the RSA and for “hyperlordotic maldistribution”, “moderate hypolordotic maldistribution”, and “severe hypolordotic maldistribution” patients for the LDI. For the LDI subdomain specifically, there was a significantly higher prevalence of post-operative moderate hypolordotic maldistributions and severe hypolordotic maldistributions in patients who underwent higher PSOs (L2 and L3) compared to lower PSOs (L4 and L5), which was the converse to the relative distributions pre-operatively.

### Revisions and reoperations

Data on mechanical complications and revisions are presented in Table 5. The overall reoperation rate for the entire cohort was 14%. There was a nonsignificant trend toward more proximal PSO levels having higher reoperation rates (L2-14%; L3-19%, respectively) compared to lower PSO levels (L4-10%; L5-0%) (*p* = 0.12). For the entire cohort, 17.1% patients had mechanical complications, with 11% having rod fractures and 6% having proximal junctional pathology requiring revisions. No significant difference in rates of mechanical complications was found between patients that were considered “moderately disproportioned” (15.4%) and “severely disproportioned” (12.8%) (*p* = 0.83). Average post-operative GAP scores were not different for patients who did and those who did not undergo revision for rod fractures for the entire cohort as well as for individual PSO levels. Average post-operative GAP scores were not different for patients who did and those who did not have revisions for PJK/PJF for the entire cohort as well as for individual PSO levels. Time to revisions for PJK/PJF were not significantly different between the 4 groups. For rod fractures, more proximal PSOs (L2-14%; L3-16%) had higher rates compared to more distal PSOs (L4-7%; L5-0%) (*p* = 0.09). Time to revision for rod fractures was significantly shorter for the L3 PSO cohort compared to L2 and L4 PSOs (*p* = 0.003).

**Table 5:** Revision operations incidences and indications

	Total (*n* = 152)	L2 PSO (*n* = 7)	L3 PSO (*n* = 62)	L4 PSO (*n* = 69)	L5 PSO (*n* = 14)	p*
Reoperations (All)	21 (14%)	2 (14%)	12 (19%)	7 (10%)	0 (0%)	0.12
*Mechanical failures*						
PJK/PJF	9 (6%)	2 (14%)	2 (3%)	5 (7%)	0 (0%)	0.039
Proportioned	0 (0%)	0 (0%)	0 (0%)	0 (0%)	0 (0%)	
Moderately disproportioned	2 (22%)	1 (50%)	1 (50%)	0 (0%)	0 (0%)	
Severely disproportioned	7 (78%)	1 (50%)	1 (50%)	5 (100%)	0 (0%)	
Post-op GAP score (avg ± SD)	8.2 ± 2.0	7.5 ± 3.5	7.5 ± 2.1	8.8 ± 1.6	N/A	0.68
Rod fractures	17 (11%)	2 (14%)	10 (16%)	5 (7%)	0 (0%)	0.09
Proportioned	0 (0%)	0 (0%)	0 (0%)	0 (0%)	0 (0%)	
Moderately disproportioned	9 (53%)	1 (50%)	6 (60%)	2 (40%)	0 (0%)	
Severely disproportioned	8 (47%)	1 (50%)	4 (40%)	3 (60%)	0 (0%)	
Post-op GAP score (avg ± SD)	6.8 ± 2.0	7.5 ± 3.5	6.6 ± 1.7	7.0 ± 2.5	N/A	0.84
*Time to revision (months)*						
PJK/PJF	30.2 ± 20.5	42.4 ± 12.7	28.6 ± 26.5	25.9 ± 22.9	n/a	0.69
Rod fractures	26.6 ± 19.7	51.0 ± 0.4	15.0 ± 11.5	40.2 ± 19.7	n/a	0.003

PSO = pedicle subtraction osteotomy; L = lumbar; PJK = proximal junctional kyphosis; PJF = proximal junctional failure; N/A = not available; GAP = Global Alignment and Proportion

*Data compared between L2, L3, and L4 only, as L5 did not have sufficient number of patients

Comparison of revision operations and incidences by L-PSO level and GAP alignment categorization L1PA and T4-L1 PA mismatch values are presented in Table 6. Comparing the four different PSO level cohorts regarding mechanical complications, rates of revision operations for PJK/PJF were found to be significantly higher in patients who underwent L2 PSOs (14%) compared to more distal PSOs (L3-3%; L4-7%; L5-0%). For the entire cohort as well as within each L-PSO level group, there were no significant differences in severity subcategory of GAP malalignment between those patients who did and did not develop mechanical complications (PJK/PJF or rod fractures). However, for the entire cohort as well as for L4 PSOs, patients who developed PJK/PJF (*n* = 9) had significantly higher post-op T4-L1 PA mismatch values compared to those who did not develop PJK/PJF. This finding was also seen for L3 PSOs [T4-L1 PA: 5.5 ± 3.5 (PJK/PJF) *v.* 1.2 ± 4.5 (no PJK/PJF)] with the difference approaching statistical significance (*p* = 0.09). Additionally, T4-L1 PA values were significantly higher for patients who developed rod fractures in the L3 and L4 PSO groups compared to those who did not develop rod fractures in their respective groups. Other than being significantly higher for L3 PSO patients who developed rod fractures, post-op L1 PA values were similar between patients who did and did not develop mechanical complications in the other PSO level groups.

**Table 6:** Comparison of revision operations and incidences by L-PSO level and GAP alignment categorization

	PJK/PJF		*p*	Rod fractures		*p*
	Yes	No		Yes	No	
Total (*n* = 152)	9 (6%)	143 (94%)	0.41	17 (11%)	135 (89%)	0.64
Proportioned	0 (0%)	1 (1%)		0 (0%)	1 (1%)	
Moderately disproportioned	2 (22%)	63 (44%)		9 (53%)	56 (41%)	
Severely disproportioned	7 (78%)	79 (55%)		8 (47%)	78 (58%)	
Post-op GAP score (avg ± SD)	8.2 ± 2.0	7.0 ± 2.2	0.13	6.8 ± 2.0	7.2 ± 2.3	0.57
Post-op L1 PA	16.2 ± 6.2	18.4 ± 9.1	0.24	16.8 ± 8.0	18.5 ± 9.1	0.24
Post-op T4-L1 PA	6.8 ± 5.3	2.3 ± 4.2	< 0.01	2.1 ± 6.6	2.6 ± 4.0	0.32
L2 (*n* = 7)	2 (14%)	5 (86%)	0.43	2 (14%)	5 (86%)	0.43
Proportioned	0 (0%)	0 (0%)		0 (0%)	0 (0%)	
Moderately disproportioned	1 (50%)	4 (80%)		1 (50%)	4 (80%)	
Severely disproportioned	1 (50%)	1 (20%)		1 (50%)	1 (20%)	
Post-op GAP score (avg ± SD)	7.5 ± 3.5	5.8 ± 1.5	0.37	7.5 ± 3.5	5.8 ± 1.5	0.37
Post-op L1 PA	9.5 ± 9.2	14.2 ± 5.8	0.22	9.5 ± 9.2	14.2 ± 5.8	0.22
Post-op T4-L1 PA	4 ± 2.8	1.2 ± 3.3	0.18	4 ± 2.8	1.2 ± 3.3	0.18
L3 (*n* = 62)	2 (3%)	60 (97%)	0.96	10 (16%)	52 (84%)	0.42
Proportioned	0 (0%)	0 (0%)		0 (0%)	0 (0%)	
Moderately disproportioned	1 (50%)	29 (48%)		6 (60%)	24 (46%)	
Severely disproportioned	1 (50%)	31 (52%)		4 (40%)	28 (54%)	
Post-op GAP score (avg ± SD)	7.5 ± 2.1	6.8 ± 2.2	0.65	6.6 ± 1.7	6.8 ± 2.3	0.75
Post-op L1 PA	13 ± 2.8	14.7 ± 6.9	0.37	20.1 ± 8.9	13.9 ± 6.3	0.01
Post-op T4-L1 PA	5.5 ± 3.5	1.2 ± 4.5	0.09	− 1.4 ± 5.8	1.9 ± 4.1	0.03
L4 (*n* = 69)	5 (7%)	64 (93%)	0.16	5 (7%)	64 (93%)	0.96
Proportioned	0 (0%)	1 (2%)		0 (0%)	1 (2%)	
Moderately disproportioned	0 (0%)	27 (42%)		2 (40%)	25 (39%)	
Severely disproportioned	5 (100%)	36 (56%)		3 (60%)	38 (59%)	
Post-op GAP score (avg ± SD)	8.8 ± 1.6	7.3 ± 2.4	0.19	7.0 ± 2.5	7.5 ± 2.4	0.67
Post-op L1 PA	20.2 ± 2.6	21.3 ± 8.8	0.4	16.6 ± 4.9	21.6 ± 8.6	0.1
Post-op T4-L1 PA	8.4 ± 6.6	3.1 ± 3.8	< 0.01	8.8 ± 4.4	3.0 ± 4.0	< 0.01
L5 (*n* = 14)	0 (0%)	14 (100%)	N/A	0 (0%)	14 (100%)	N/A
Proportioned	0 (0%)	0 (0%)		0 (0%)	0 (0%)	
Moderately disproportioned	0 (0%)	3 (21%)		0 (0%)	3 (21%)	
Severely disproportioned	0 (0%)	11 (79%)		0 (0%)	11 (79%)	
Post-op GAP score (avg ± SD)	N/A	7.3 ± 1.4	N/A	N/A	7.3 ± 1.4	N/A
Post-op L1 PA	N/A	23.4 ± 12.6	N/A	N/A	23.4 ± 12.6	N/A
Post-op T4-L1 PA	N/A	4.1 ± 4.0	N/A	N/A	4.1 ± 4.0	N/A

PSO = pedicle subtraction osteotomy; L = lumbar; PJK = proximal junctional kyphosis; PJF = proximal junctional failure; N/A = not available; GAP = Global Alignment and Proportion; PA = pelvic angle

## Discussion

Lumbar pedicle subtraction osteotomy (L-PSO) is a powerful surgical tool to correct sagittal spinal deformity. Their immediate and long-term utility hinge on several factors, including achieving union across the osteotomy site, preservation of neural function, magnitude of segmental correction, and restoration of appropriate spinal shape. In an attempt to improve our field’s understanding of the optimal level at which to perform a L-PSO, we have presented a retrospective cohort analysis comparing the effects on the GAP score of 4 different L-PSO levels. This study has the following 5 major findings: (1) L-PSOs, irrespective of level, improve GAP scores and associated subdomains’ scores and descriptive alignment categories; (2) despite these improvements in GAP score, residual disproportionate post-operative alignment was observed for all L-PSO levels; (3) lower PSOs (L4 and L5) improved L4-S1 lordosis and the lumbar distribution index to a greater extent than more proximal PSOs (L2 and L3); (5) more distal PSOs (L4 and L5) had lower rates of revision operations for mechanical complications, particularly for PJF, compared to more proximal PSOs (L2 and L3); and (5) average post-operative GAP scores were not different for patients who did and those who did not have revisions for mechanical failures for the entire cohort as well as for individual PSO levels. The results of this study complement and add a unique perspective to the current literature on the differential effects of L-PSO level on sagittal plane realignment.

Identifying the ideal location at which a L-PSO should be performed to optimize sagittal alignment has been a topic of much interest, but with limited comparative data. In 2011, Lafage et al. were the first to compare sagittal realignment based on different PSO levels (L1 vs. L2 vs. L3 vs. L4) [9]. Specifically, they reported in their multi-center study that: (1) there was no significant difference in focal correction achieved by level of L-PSO (L1: 24°; L2: 24°; L3: 25°; L4: 22°) and (2) all patients had significant improvements in all sagittal alignment parameters, including increases in lumbar lordosis and thoracic kyphosis as well as decreases in C7-S1 sagittal vertical axis (SVA), T1 spinopelvic inclination, and PT [9]. More recently, Zavras et al. in 2023 published a retrospective cohort analysis of 53 patients who underwent L-PSOs at different levels [L1: *n* = 3 (5.7%); L2: *n* = 6 (11.3%), L3: (39.6%), L4: (39.6%), L5: 3.8%] in which it was reported that the degree of PSO segmental correction was similarly distributed across L-PSO levels [23]. Our data on standard radiographic alignment parameters and degree of segmental correction by PSO level are in concordance with these two prior studies [9, 23]. Uniquely, our study also evaluated L1 PA, T4 PA, and T4-L1 PA mismatch values relative to level of L-PSO. In this realm we found that L1PA and T4PA angles were significantly higher pre-operatively and remained significantly higher post-operatively in patients who underwent more distal lumbar PSOs (L4 and L5) compared to more proximal PSOs (L2 and L3) despite L4 and L5 lumbar PSOs providing significantly greater improvements in L1PA. Despite these findings, note that L5 PSOs in our study provided less L1-S1 lordosis gain than the proximal PSOs, the etiology of which may be multifactorial. One possible reason for this difference was that the patients who underwent L5 PSOs had relatively greater L1-S1 preoperatively possibly as a result of hyperextension of the upper lumbar spine (L1-4) as a compensation for deformities emanating from the distal lumbar spine (L4-S1). In turn, after the L5 PSO, the upper lumbar spine’s relative hyperextension decreased resulting in more lordosis emanating from the distal lumbar spine and less from the upper lumbar spine. While an overall increase, the relative change was less pronounced than more proximal PSOs, which may not have had as much of a compensatory lordosis present preoperatively. Additionally, angular correction provided by L5 PSO’s was more variable (i.e., greater range and standard deviation), which may also have resulted in less average overall L1-S1 improvement. While other factors may also have contributed to this phenomenon, the relatively less L1-S1 lordosis gain from L5 PSOs may have been the explanation behind the lack of difference in global alignment and overall correction from L5 PSOs relative to the more proximal PSOs.

In addition to restoration of regional and global sagittal alignment, restoration of appropriate spinal shape and distribution of lumbar lordosis in relation to pelvic incidence and pelvic orientation are important. In a landmark article by Pesenti et al. that evaluated lumbar lordosis in a normative patient population, it was demonstrated that upper lumbar lordosis (L1-L4) varied based on PI magnitude, while L4-S1 lordosis was relatively homogeneous (average 35°–40°) for all patients, irrespective of PI magnitude [33]. Restoration of appropriate L4-S1 lordosis has been demonstrated in several clinical investigations to portend better outcomes. Herrington et al. and Manoharan et al. concluded that a loss of L4-S1 lordosis following short-segment lower lumbar fusions may play a significant role in the development of adjacent segment degeneration and malignment and associated reoperation [34, 35]. These findings are supported by a cadaveric study in which decreased L4-S1 lordosis resulted in increased load, shearing, and lordosis at adjacent levels [36]. Thus, insufficient restoration of L4-S1 lordosis or LDI increases the risk of regional lumbar and global spinal malalignment and mechanical complications post-operatively, as alluded to by Huec et al. [37] and Zheng et al. [38]. As anticipated, we found that more distal PSOs (L4 and L5) improved L4-S1 lordosis to a greater extent than more proximal PSOs (L2 and L3). This, in turn, was reflected in restoring better lumbar shape, as evidenced by significantly higher post-operative GAP LDI scores for L4 and L5 PSOs relative to L2 and L3 PSOs. This may also partially explain the findings of significantly greater improvements in L1PA values after more distal lumbar PSOs (L4 and L5) compared to more proximal (L2 and L3) PSOs.

To our knowledge, only one prior study has presented data on GAP scores following L-PSOs. In 2023, Yahanda et al. presented a case series of 40 patients who underwent L-PSO at different levels [L1: *n* = 1 (2.5%); L2: *n* = 7 (17%), L3: *n* = 29 (72.5%), L4: *n* = 7 (17.5%)] [24]. While they reported a mean PSO angle correction of 28.7 ± 7.6°, significant improvements in several sagittal alignment parameters (C7-S1 SVA, PI-LL mismatch, PT), and significant improvements in total GAP score, no data were presented on the GAP’s subdomains and no comparative analyses of the radiographic alignment parameters and the GAP score and its subdomains were performed with respect to different L-PSO level [24]. Nevertheless, there are several noteworthy comparisons between our study and that of Yahanda et al. First, we both found that while average total GAP scores significantly improved after surgery [Yahanda: 10 ± 2 (preop) to 8 ± 2 (postop) *v.* This study: 9.8 ± 2.8 (preop) to 7.1 ± 2.2 (postop)], the vast majority of post-operative patients remained “disproportioned” (Yahanda: 100% “severely disproportioned” *v.* This study: 43% “moderately disproportioned” and 57% “severely disproportioned”). Yahanda et al*.* had astutely speculated in their study that, “the inability to bring the average patient from “severely disproportioned” to “moderately disproportioned” is likely due to the high fraction of PSOs that were performed at L3 in previously fused spines, which tempered the effect that the PSO had on correcting low lumbar lordosis. Changing the distribution of lordosis by performing a PSO at L4 or L5 versus L3 may produce a greater degree of sagittal SVA correction [24]. While we did observe patients convert out of a “severely disproportioned” category for L4 PSOs and L5 PSOs, this was also observed for L2 PSOs and L3 PSOs. That none were fully restored to “proportioned” alignment postoperatively is likely attributable to the focal angular correction of L-PSOs at only one level in a previously fused spine in which all levels are segmentally misaligned, as opposed to harmonious correction of lordosis across multiple levels (L1-S1) that may be achieved with intradiscal surgical techniques in unfused or partially fused lumbar spines [2]. Despite the GAP scores suggesting persistent malalignment, the average values for post-operative T4-L1 PA mismatch for all L-PSO groups were within the 4^0^ threshold proposed by Hills et al. [39, 40], suggesting overall spinal shape and balance between the thoracic and lumbar spines were relatively “acceptable”. However, the wide ranges for our T4-L1 PA mismatch values, as evidenced by large standard deviations, suggest considerable variation in spinal shape and the observed persistently “malaligned” GAP scores following L-PSO, irrespective of PSO level.

With respect to mechanical complications, we found a 17.1% rate of revision surgery for proximal junctional pathology (PJK/PJF) (11%) and rod fractures (6%). This complication rate is strikingly lower than that predicted by the average post-operative GAP score of 7.1 ± 2.2 and the vast majority (90%) of post-operative patients being “disproportioned”, which was a similar finding to that in the aforementioned study by Yahanda et al. (rod fracture—5%; PJK/PJF—7.5%) [24]. Also in contrast to the original study that validated the GAP score [28], we found similarly lower rates of mechanical complications for “moderately disproportioned” and “severely disproportioned” patients. Comparing the 4 different PSO level cohorts regarding mechanical complications, rates of revision operations for PJK/PJF were found to be significantly higher in patients who underwent L2 PSOs (14%) compared to more distal PSOs (L3-3%; L4-7%; L5-0%). This is in contrast to Zavras et al*.* who noted similar rates of proximal junctional pathology between L-PSO levels (L1: 33.3%; L2: 16.7%; L3: 4.8%; L4: 23.8%; L5: 50%). Additionally, more proximal PSOs had higher rates of rod fractures compared to more distal PSOs (L2-14%; L3-16%; L4-7%; L5-0%) [23]. This same relative trend was also found in the aforementioned study by Zavras et al. (L2: 50%; L3: 38.1%; L4: 19%; L5: 0%) [23]. These data may be interpreted and lend credence to the notion that more distal L-PSOs decrease mechanical complications relative to more proximal L-PSOs as the result of better LDI scores, irrespective of total GAP score.

While total GAP scores and GAP malalignment severity subcategories did not differ between patients in any L-PSO group who sustained mechanical complications and those who did not, there were significant differences between T4-L1 PA mismatch values between those who did and did not develop mechanical complications for certain PSO levels. Specifically, for the entire cohort as well as for L4 PSO patients, those who developed PJK/PJF had significantly higher post-op T4-L1 PA mismatch values compared to those who did not develop PJK/PJF. This finding was also seen for L3 PSO patients, with the difference approaching significance. Additionally, T4-L1 PA values were significantly higher for patients who developed rod fractures in the L3 and L4 PSO groups compared to those who did not develop rod fractures in their respective groups. These findings are consistent with prior literature demonstrating that deviations in T4-L1 PA mismatch portend higher risk for development of post-operative mechanical complications [39].

While components of sagittal alignment may contribute to these findings, etiology of mechanical failures is highly heterogeneous and includes a variety of other biologic factors (bone density, frailty, sarcopenia), surgical factors (rod shape, rod bending, rod material, number of rods), and other mechanical and extraspinal alignment factors (i.e., BMI, hip flexion contractures). Another factor that is important for dictating union and rod fracture across a L-PSO is use of interbody support adjacent to the L-PSO site [16], which was used in all disks caudal to the PSO if no prior interbody support was present at that level. No interbodies were used within the PSO site or at the cranial supra-adjacent disk to the PSO site given all PSOs were considered Schwab Type IV osteotomies. In addition to interbody support, number of rods may dictate rod failure rates. Note that in our study the more proximal PSOs (L2 and L3) had significantly higher usage of only 2-rod constructs, while more distal PSOs (L4 and L5) had significantly higher usage of multi-rod (≥ 4) constructs, which could explain the latter’s lower rate of rod failures. As such, differences in rod construct complexity (i.e., greater number of rods and dual iliac fixation in distal PSOs) may confound the association between PSO level and mechanical failure rates. Given these additional factors were neither assessed nor controlled for, we cannot conclude that our observed differences in PJK/PJF and rod fracture rates are solely secondary to the GAP score and associated spinal radiographic alignment parameters.

### Limitations

The findings of this study should be considered within the context of its limitations. The retrospective nature of this study introduces potential bias and prevents against comprehensive evaluation of all factors that may influence the evaluated outcome measures. Additionally, this study only includes patients from a single-center, which may limit its external validity to other institutions or patient populations. Additionally, as we excluded patients with instrumented fusions that extended to the upper thoracic spine so as to disconnect the influence of thoracic instrumentation/correction from the sagittal spinal parameters, our findings may not be generalizable to upper-thoracic to pelvis operations for ASD. While the average follow-up was considerably robust, it was also quite heterogeneous and may not capture longer-term outcomes or complications associated with L-PSOs. Furthermore, the average follow-up was significantly different between the four groups, with L2 and L3 PSOs having significantly longer follow-up than L4 and L5 PSOs. There were also significant differences in number of rods and number of points of pelvic fixation between the PSO groups with L4 and L5 PSOs having significantly more constructs consisting of multiple rods (≥ 4) and dual iliac screw fixation bilaterally. These variations reflect the field’s evolving understanding of the importance of multiple rods crossing L-PSOs to decrease rod fracture rates, the “ideal location” to perform a PSO, and its evolving appreciation for restoration of appropriate spinal shape and low lumbar lordosis (L4-S1) with the goal to minimize mechanical complications. A further limitation is the relatively small sample size of 152 patients with seven L2 PSO patients and fourteen L5 PSO patients, which may limit the statistical power to detect significant differences or associations. However, our sample sizes are comparable or greater than the aforementioned investigations on this topic. Lastly, there may be variability in measurement accuracy of radiographic spinal alignment parameters despite having one person conduct all measurements to ensure consistency, standardization, and reduce bias. Despite these limitations, the results of this study may be considered a unique addition to the expansive literature on sagittal spinal realignment from L-PSOs. Additionally, we anticipate these data and thoughts will continue to spark debate and discussion and encourage additional multi-center studies to explore the impact of L-PSO level selection on sagittal alignment scoring systems and classifications (i.e., GAP score, Roussouly), mechanical complications, and patient-reported outcomes.

## Conclusions

In adults undergoing lumbar PSOs and revision multi-level thoracolumbar instrumented fusions from the TL junction to the pelvis, GAP scores significantly improved irrespective of the level of L-PSO. While residual disproportionate post-operative alignment was observed for all L-PSO levels, more distal PSOs (L4 and L5) improved L4-S1 lordosis to a greater extent than more proximal PSOs (L2 and L3), which in turn resulted in a better LDI. Although more distal lumbar PSOs had lower rates of revision operations for mechanical complications, particularly for proximal junctional kyphosis/failure, other patient and surgical factors also likely played a role in the observed rates of mechanical failures. Additional future studies investigating the long-term impact of different PSO levels on patient-reported outcomes would provide valuable insights into the relative clinical utility of L-PSOs performed at different levels.

## Data Availability

The data that support the findings of this study are available from the corresponding author, AAT, upon reasonable request.
